# Comparative analysis of morabine grasshopper genomes reveals highly abundant transposable elements and rapidly proliferating satellite DNA repeats

**DOI:** 10.1186/s12915-020-00925-x

**Published:** 2020-12-21

**Authors:** Octavio M. Palacios-Gimenez, Julia Koelman, Marc Palmada-Flores, Tessa M. Bradford, Karl K. Jones, Steven J. B. Cooper, Takeshi Kawakami, Alexander Suh

**Affiliations:** 1 Department of Ecology and Genetics – Evolutionary Biology, Evolutionary Biology Centre, Uppsala University, SE-752 36 Uppsala, Sweden; 2 Department of Organismal Biology – Systematic Biology, Evolutionary Biology Centre, Uppsala University, SE-752 36 Uppsala, Sweden; 3 Evolutionary Biology Unit, South Australian Museum, Adelaide, SA 5000 Australia; 4 School of Biological Sciences and Australian Centre for Evolutionary Biology and Biodiversity, The University of Adelaide, Adelaide, SA 5005 Australia; 5 Embark Veterinary, Inc., Boston, MA USA; 6 School of Biological Sciences, University of East Anglia, Norwich Research Park, Norwich, NR4 7TU UK

## Abstract

**Background:**

Repetitive DNA sequences, including transposable elements (TEs) and tandemly repeated satellite DNA (satDNAs), collectively called the “repeatome”, are found in high proportion in organisms across the Tree of Life. Grasshoppers have large genomes, averaging 9 Gb, that contain a high proportion of repetitive DNA, which has hampered progress in assembling reference genomes. Here we combined linked-read genomics with transcriptomics to assemble, characterize, and compare the structure of repetitive DNA sequences in four chromosomal races of the morabine grasshopper *Vandiemenella viatica* species complex and determine their contribution to genome evolution.

**Results:**

We obtained linked-read genome assemblies of 2.73–3.27 Gb from estimated genome sizes of 4.26–5.07 Gb DNA per haploid genome of the four chromosomal races of *V. viatica*. These constitute the third largest insect genomes assembled so far. Combining complementary annotation tools and manual curation, we found a large diversity of TEs and satDNAs, constituting 66 to 75% per genome assembly. A comparison of sequence divergence within the TE classes revealed massive accumulation of recent TEs in all four races (314–463 Mb per assembly), indicating that their large genome sizes are likely due to similar rates of TE accumulation. Transcriptome sequencing showed more biased TE expression in reproductive tissues than somatic tissues, implying permissive transcription in gametogenesis. Out of 129 satDNA families, 102 satDNA families were shared among the four chromosomal races, which likely represent a diversity of satDNA families in the ancestor of the *V. viatica* chromosomal races. Notably, 50 of these shared satDNA families underwent differential proliferation since the recent diversification of the *V. viatica* species complex.

**Conclusion:**

This in-depth annotation of the repeatome in morabine grasshoppers provided new insights into the genome evolution of Orthoptera. Our TEs analysis revealed a massive recent accumulation of TEs equivalent to the size of entire *Drosophila* genomes, which likely explains the large genome sizes in grasshoppers. Despite an overall high similarity of the TE and satDNA diversity between races, the patterns of TE expression and satDNA proliferation suggest rapid evolution of grasshopper genomes on recent timescales.

**Supplementary information:**

**Supplementary information** accompanies this paper at 10.1186/s12915-020-00925-x.

## Background

Eukaryotic genomes exhibit repetitive DNA sequences represented by interspersed transposable elements (TEs) and tandem repeats (TRs; e.g., satellite DNA; satDNA), collectively known as the “repeatome” [1]*.* TEs occupy a large fraction of genomes in organisms throughout the tree of life [2]. The ubiquity of TEs is driven by the ability to either copy and paste themselves with an RNA intermediate step (retrotransposons, class I) or to cut and paste themselves (most DNA transposons, class II) within the genome of the host organism [3–5]*.* Each TE class can be further categorized by elements that encode protein products required for transposition (autonomous) and those that only contain the sequences (non-autonomous) necessary for trans-recognition by the transposition machinery of an autonomous counterpart [6]*.* Class I elements comprise short interspersed nuclear element (SINE), long interspersed nuclear element (LINE), and long terminal repeat (LTR) retrotransposon. Class II elements comprise DNA transposons like terminal inverted repeat (TIR) elements, Crypton, Helitron, and Maverick [7]. The mechanism of transposition allows TEs to invade the genome in a parasitic way without general advantage to the individual carrying them [3] but with potentially deleterious effects on their host by promoting ectopic recombination, mediating chromosomal rearrangements, and disrupting coding sequences [8–10]*.*

Another type of repetitive element widely distributed in eukaryotic genomes is satDNA. It consists of non-coding repetitive DNA that is tandemly arranged and largely represented in the centromeric and pericentromeric heterochromatin of most eukaryotic genomes [11–13]. satDNA evolution is influenced by several mechanisms of non-reciprocal genetic exchange such as unequal crossing over, intra-strand homologous recombination, gene conversion, rolling-circle replication, and transposition [11, 14–17]. These mechanisms can gradually increase the copy number of a new sequence variant within a satDNA family across the genomes of a sexual population [11, 14–16, 18, 19]. Sequences within a satDNA family undergo concerted evolution as repeat exchanges occur both within and between members of the satDNA family by non-reciprocal genetic transfers between homologous and sometimes non-homologous chromosomes [14, 20]. This results in frequent homogenization of repeats between copies within species and also between repeat copies located on a same chromosome than between different chromosomes [14, 20]. At the same time, the primary sequence of satDNAs usually mutates quickly, and this rapid satDNA turnover leads to distinct composition and genomic distribution of satDNAs between strains, populations, subspecies, or species [11, 15, 18, 19, 21, 22]*.* However, there have been reports of satDNA sequence conservation across extraordinarily long evolutionary periods in bivalve mollusks [23–25], ants [26], and Bovidae [27]*.* The library hypothesis proposes that species do not entirely lose or gain certain lineages of satDNAs, but, instead, related species share a common collection of satDNAs that may independently increase or decrease in their copy numbers during or after speciation [28, 29]. Sequence divergence as the outcome of reproductive isolation might then lead to a formation of species-specific profiles of satDNA sequence variants [30, 31]. For grasshoppers and crickets (Orthoptera), high-throughput sequencing analyses detected 316 satDNA families in ~ 20 species [12, 21, 32–37]*.* Because of the large divergence time between these Orthopteran species (73–224 million years, Myr) [38], none of these 316 satDNA families showed interspecific homology, except for partial sequence homology within satDNAs of *Gryllus* cricket species [32] (unknown divergence time) and within *Schistocerca* grasshopper species [21] with divergence time < 8 Myr [39]. Because homology between satDNAs often cannot be detected between distantly related species due to rapid sequence divergence, an explicit examination of the library hypothesis requires well-annotated genomes of closely related species.

The repeatome is involved in the processes of sex chromosome differentiation [11, 40–43]. Both plants and animals have accumulated TEs and satDNAs in the non-recombining regions of Y and W chromosomes [11, 40, 42–46]*.* Studies on neo-Y chromosomes of several *Drosophila* species proposed that the first steps of Y chromosome degeneration are driven by accumulation of TEs and satDNAs [41, 45]. The non-recombining parts of the Y or W chromosomes may thus expand by repeat accumulation and heterochromatinization [41, 45]. Moreover, transcription of satDNAs (satRNAs) may have a critical role in centromere function, chromatin silencing, heterochromatin formation, chromatin modulation, and upregulation of X-linked genes in chromosome dosage compensation [47–50]. It has been proposed that interspecific incompatibilities in hybrids between satRNAs and specific proteins involved in centromere function can contribute to genome divergence and the speciation process [30, 49, 51–53]*.*

Grasshoppers generally have large genomes (9 Gb on average, minimum 1.5 Gb and maximum 16.6 Gb [54]) likely because of large amounts of repetitive DNA [55, 56]. They provide ample opportunities to investigate the influence of the repeatome on karyotype evolution because grasshoppers are also karyotypically divergent between closely related species [57–59]*.* However, comparative genomic studies in grasshoppers have been hampered by their large genome sizes [55, 56]. The Australian morabine (Morabinae) grasshopper of the genus *Vandiemenella* (hereafter referred to as the viatica species group) is a relatively young species complex with estimated divergence time < 0.5–3.1 Myr based on a mitochondrial marker [58] and is karyotypically diverse [58–60]*.* It currently contains two nominal species (*Vandiemenella pichirichi* and *Vandiemenella viatica*) and five provisional species (P24, P25, P45b, P45c, and P50) differentiated by one or more chromosomal rearrangements [61, 62]. Earlier cytogenetics studies hypothesized that the chromosomal race viatica19 (2*n* = 19, X0 male) is most closely resembling the ancestral karyotype of the *viatica* species group [59, 60]. Subsequent sequential chromosomal rearrangements, including centric fusions, fissions, and inversions, resulted in the formation of the present taxa [59, 60, 63]. Notably, neo-sex chromosomes in the *viatica* species group evolved three times independently through recent fusions of the ancestral X chromosome with a different autosome each time (P24X0/XY, P25X0/XY, and P45bX0/XY races) [59, 60]. Given that repetitive sequences can play critical roles in the evolution of genome structure and function, a comprehensive analysis of TEs and satDNAs of these grasshopper genomes is essential for the understanding of the genome structure and chromosomal evolution of the *viatica* species group.

Here, we characterized TEs and satDNAs in four chromosomal races of the *viatica* species group, P24X0, P24XY, P45bX0, and P45bXY, by generating 10X Genomics Chromium linked-read data and RNA sequencing data. We then used three complementary methods, namely homology-based, structure-based, and de novo approaches, for annotating the TE and satDNA fraction of the genomic reads and assemblies. By comparing sequence divergence within the TE classes, we identified the temporal dynamics of TE accumulation. We identified transcriptional activity of many subfamilies of TEs in three tissues of these grasshoppers, showing that the TE expression levels vary greatly among TEs, tissues, and sexes. We also provided evidences that satDNAs expanded and contracted in their genomic copy numbers at different time points since the divergence of the chromosomal races.

## Results

### Genome assembly

**Fig. 1:**
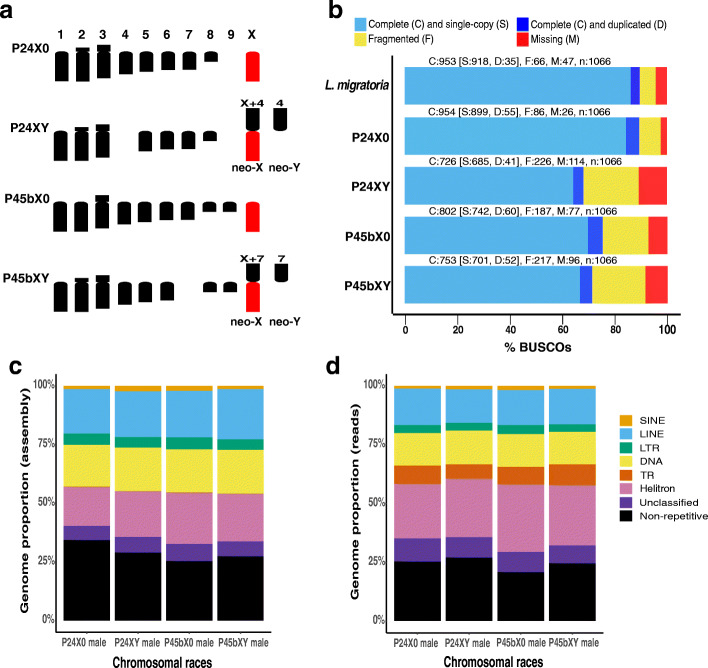
Overview of the karyotype, BUSCO assembly assessment, and repeat composition across four chromosomal races of the *viatica* species group*.* **a** Karyotypes of the four chromosomal races. Chromosomes are aligned based on centromere position. Only haploid sets of chromosomes in males are shown. Two independent emergences of neo-XY sex chromosomes via fusions between the ancestral X chromosome (red) and one of the autosomes (black) are highlighted. **b** BUSCO completeness assessment for genome assembly quality control. Color-coded bar plot showing the proportions of BUSCO genes classified as complete, complete single-copy, complete duplicated, fragmented, and missing. **c**, **d** Color-coded bar plot illustrating the proportions of major TE groups, unclassified repeats, tandem repeats (TRs), and non-repetitive regions detected in the assembled genomes (**c**) and in the sequenced reads (**d**). The major TE groups included manually curated RepeatModeler consensus sequences and Helitrons identified by HelitronScanner

We sequenced male genomes of four chromosomal races representing two pairs of karyotypes with and without neo-sex chromosomes (Fig. 1a) using 10X Genomics Chromium linked-read libraries with 1577–1883 million paired-end reads per library. Average input molecule lengths between races ranged from 17.44 to 56.11 kb. By using Supernova 2.1.0 [64], we obtained genome assemblies with the following sizes: 3.02 Gb in P24X0, 2.73 Gb in P24XY, 3.27 Gb in P45bX0, and 2.94 Gb in P45bXY. The contig N50 for genome assemblies ranged from 29.11 to 35.69 kb, and the scaffold N50 ranged from 34.85 to 316.69 kb. Chromosomal races with larger inferred genome assembly sizes tended to have more fragmented genomes (Table 1). Using BUSCO v3 [65] with the Arthropoda dataset, the P24X0 assembly had a similar proportion of complete single-copy orthologs (90%) as the Illumina assembly of the migratory locust *Locusta migratoria* (Acrididae, Oedipodinae) (89%) [55]. The P24XY, P45bX0, and P45bXY assemblies had lower numbers of single-copy orthologs (65–70%) than P24X0 and *L. migratoria*. The BUSCO scores also indicated that the fractions of genes that were entirely missing were lower in the P24X0 race than the other chromosomal races (P24XY, P45bX0, and P45bXY) and the *L. migratoria* genome assembly (Fig. 1b).

**Table 1:** Assembly statistics across the four chromosomal races of the *viatica* species group. Pseudohaploid assemblies were analyzed without any size cutoff for contig or scaffold length

	P24X0 male	P24XY male	P45bX0 male	P45bXY male	
Total millions of reads (2 × 150 bp)	1883	1582	1759	1577	10X Genomics Chromium linked reads
Molecule length (kb)	41.18	17.44	56.11	30.44	Average input molecule lengths
Longest scaffold (kb)	44.43	100.68	88.04	99.29	
Scaffold N50 (kb)	316.69	34.85	51.83	36.43	N50 scaffold size
Edge N50 (kb)	8.44	6.24	8.24	6.73	N50 edge size
Contig N50 (kb)	35.69	26.94	33.34	29.11	N50 contig size
Phaseblock N50 (kb)	651.54	50.13	115.56	53.56	N50 phase block size
Assembly GC content (%)	37.99	37.93	38.09	38.01	
Reads aligned to reference (%)	81.12	78.47	80.08	76.10	
Assembled genome size (Gb)	3.02	2.73	3.27	2.94	Supernova assembler
Genome size estimation (Gb)	3.30	3.36	3.52	3.82	Supernova (computed from k-mer distributions)
	5.42	5.65	5.76	6.32	FindGSE (computed from k-mer distributions)
**Average genome size (Gb)**	**4.26**	**4.50**	**4.64**	**5.07**	Based on k-mer distributions

Owing to the incompleteness of virtually all animal genome assemblies [66, 67], genome assembly length tends to be smaller than the actual genome size. To ameliorate this underestimation, we inferred a genome size from sequenced reads directly by analyzing the frequency of k-mers using the findGSE function [68]. The measures of genome sizes varied between each pipeline used, i.e., between findGSE and the Supernova estimations computed from k-mer distributions (Table 1). We thus report average values between these k-mer-based measures as an approximation of the true genome sizes. This analysis resulted in genome size estimates of 4.26 Gb in P24X0, 4.50 Gb in P24XY, 4.64 Gb in P45bX0, and 5.07 Gb in P45bXY. To show the overall genomic complexity of the four chromosomal races, we estimated the k-mer spectrum (k-mer coverage 21.57–25.29), heterozygosity (1.85–2.91%), and repetitive content (71–80%) based on the k-mer profile (Additional file 2: Figure S1).

### Transposable element (TE) identification

We first ran RepeatModeler 1.0.8 [69] on each of the four genome assemblies to generate benchmark repeat libraries for annotating the TEs, yielding between 1361 and 1398 consensus sequences per race. Between 637 and 668 consensus sequences were initially classified as unknown by RepeatModeler in each library. To further classify these unknown repeats, we manually curated the P24X0 repeat library generated by RepeatModeler because this had the best genome assembly quality based on contig N50 and BUSCO scores. Our manual curation identified 212 new consensus sequences of TE subfamilies in the P24X0 genome (32% of the unknown repeat consensus sequences above). Next, we used the curated TE consensus sequences of P24X0 to re-classify unknown repeats in the other three libraries by homology searches in RepeatMasker 4.0.8 [70]. This classified a total of 215, 206, and 212 new TE subfamilies (~ 34% of the unknown repeat consensus sequences above) in the P24XY, P45bX0, and P45bXY genomes, respectively. From these newly identified TEs, about 2.24–2.69% of each genome assembly (83–93 subfamilies) was reclassified as LTR retrotransposons and 2.13–2.78% of each genome assembly (77–98 subfamilies) as DNA transposons. The comparison of TE landscapes, i.e., the distribution of TE-derived bp in bins of Kimura 2-parameter (K2P) distance, in raw RepeatModeler repeat libraries vs. curated libraries highlights the improvement of TE annotation by in-depth manual curation and reclassification (Additional file 2: Figure S2).

**Fig. 2:**
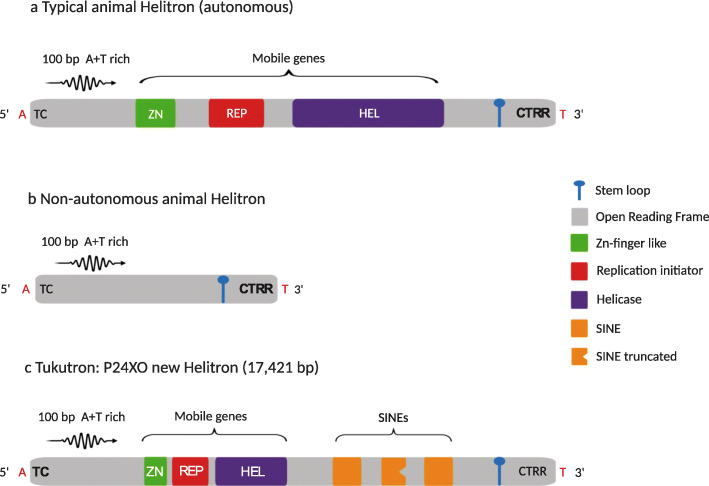
Helitron structures found in the genome assemblies of the *viatica* species group. **a** Typical autonomous animal Helitron. **b** Non-autonomous animal Helitron. **c** The new identified Helitron family identified in the P24X0 chromosomal race, named Tukutron. The new Helitron bears entire and truncated copies of short interspersed elements (SINEs) which are similar to SINEs in the *Locusta migratoria* genome (SINE2-3_Lmi in Repbase)

To further improve the TE annotation, we applied HelitronScanner 1.0 [71] to search for low-copy Helitrons that were missed by RepeatModeler. HelitronScanner and clustering of the nucleotide dataset into clusters that met a similarity threshold of 80% produced a set of non-redundant representative sequences (families) of new Helitrons, i.e., 230, 211, 286, and 221 Helitron families in the P24X0, P24XY, P45bX0, and P45bXY genomes, respectively. We identified a new family of autonomous Helitron (“Tukutron”) in the P24X0 genome (Fig. 2) in which entire and truncated SINEs were nested in its sequence. Tukutron comprises 12,053 fragments in the genome of P24X0 (0.01% of the genome). The bp percentages of all Helitron sequences in the genome assemblies were 10% (2,743,812 fragments) in P24X0, 13% (2,578,114 fragments) in P24XY, 15% (3,207,327 fragments) in P45bX0, and 13% (2,837,154 fragments) in P45bXY.

RepeatMasker analysis of respective genome assemblies with a race-specific combined repeat library (race-specific libraries from RepeatModeler, HelitronScanner, and RepeatExplorer2 combined with Repbase Arthropoda repeats; RepeatExplorer2 details below) revealed that from 66 to 75% of the assembled genomes of the *viatica* species group was composed of TEs (Fig. 1c, Table 2). The genome proportion of LINEs (18.88–21.41%) was highest among the annotated major TE groups, followed by Helitrons (16.72–21.88%), DNA transposons (17.89–18.66%), LTRs (4.39–5.19%), and SINEs (1.45–2.31%) (Table 2). Regarding superfamilies of TEs, the most abundant across the assembled genomes was Helitron (17–22%), followed by DNA/TcMar (7%–9%) and LINE/CR1 (7–10%). Since TRs were likely underrepresented in genome assemblies, the read-based RepeatExplorer2 [72–74] and NOVOplasty 3.7.2 protocols [75] were applied to detect TRs (see below). None of the TRs detected using these two approaches were recovered by the above RepeatModeler repeat libraries. The relative genomic abundance of detected TRs in the sequenced reads was then compared by sampling 4 million read pairs per library and aligning them to the aforementioned race-specific combined repeat library with RepeatMasker; this quantification was done separately in each race. The analysis revealed that 73 to 79% of the sequenced reads of the *viatica* species group was composed of repeats (Fig. 1d, Table 3) and showed that, while overall TE proportions were comparable to the assembly proportions, TRs and Helitron sequences were better represented in the read-based approach than in the assembly-based approach (Fig. 1c,d; Tables 2 and 3).

**Table 2:** Assembly-based quantification of repeats. Copy number, total base pair, and density of different classes of repeats annotated by RepeatMasker using a combined library of the RepeatModeler de novo library from each race (manual curation of the P24X0 library, used for reclassification of the three other de novo libraries), the Arthropod library from Repbase, and libraries from each race from HelitronScanner and RepeatExplorer2, across males of four chromosomal races of the *viatica* species group. *TR* tandem repeats, *GP* genome proportion (% assembly)

Repeat type	P24X0			P24XY				P45bX0			P45bXY	
	Copies	Total bp	GP	Copies	Total bp	GP	Copies	Total bp	GP	Copies	Total bp	GP
SINE	355,321	57,777,060	1.45	476,672	84,209,572	2.31	498,884	86,588,794	2.16	331,980	55,473,057	1.45
LINE	1,630,136	751,701,393	18.88	1,544,315	715,290,166	19.6	1,603,310	792,142,166	19.79	1,780,161	819,481,256	21.40
LTR	270,520	191,052,819	4.80	298,156	160,264,290	4.39	316,814	207,702,027	5.19	303,192	169,275,629	4.42
DNA	2,269,382	712,386,285	17.89	2,136,964	678,964,713	18.61	2,179,781	734,004,927	18.34	2,165,693	714,794,492	18.66
Helitron	3,194,220	663,607,802	16.72	8,654,890	708,282,512	19.45	3,887,696	878,010,926	21.88	3,569,564	782,636,309	20.45
TR	5269	790,157	0.02	3294	506,934	0.01	10,988	1,403,691	0.04	232	12,409	0.01
Unclassified	889,785	237,465,164	5.98	795,764	240,830,299	6.62	946,725	290,976,813	7.29	8,992,548	237,984,790	6.24
**Total**	**8,614,633**	**2,614,780,680**	**65.74**	**13,910,055**	**2,588,348,486**	**70.99**	**9,444,198**	**2,989,425,653**	**74.69**	**17,143,370**	**2,779,657,942**	**72.62**

**Table 3:** Read-based quantification of repeats by sampling 4 million read pairs per library. Density of different classes of  repeats annotated by RepeatMasker using a combined library of the RepeatModeler de novo library from each race (manual curation of the P24X0 library, used for reclassification of the three other de novo libraries), the Arthropod library from Repbase, and libraries from each race from HelitronScanner and RepeatExplorer2, across males of four chromosomal races of the *viatica* species group. *TR* tandem repeats, *GP* genome proportion (% reads)

Repeat type	P24X0	P24XY	P45bX0	P45bXY
	GP	GP	GP	GP
SINE	1.23	1.49	1.81	1.27
LINE	15.48	14.26	14.91	15.09
LTR	3.39	3.29	3.81	3.28
DNA	13.83	14.43	13.95	13.77
Helitron	23.17	24.84	28.73	25.67
TR	7.82	6.06	7.53	8.85
Unclassified	9.86	8.65	8.50	7.51
**Total**	**74.78**	**73.02**	**79.44**	**75.44**

### Temporal accumulation of TEs

**Fig. 3:**
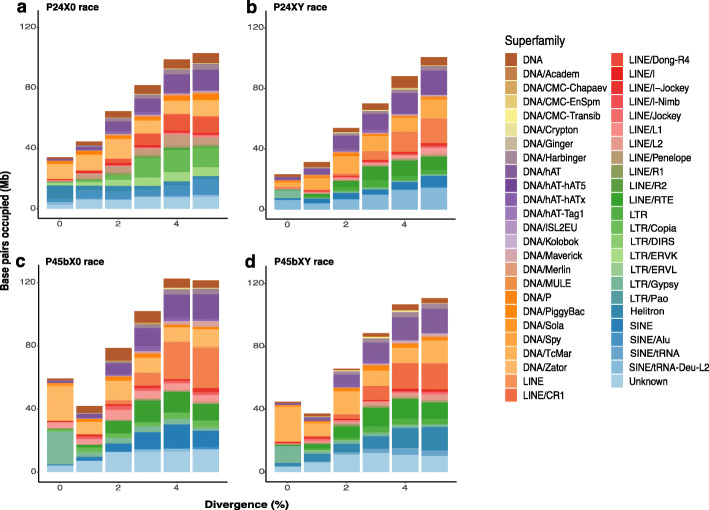
Repeat landscapes illustrating recent accumulation of 49 superfamilies of transposable elements across four chromosomal races of the *viatica* species group. **a** P24X0. **b** P24XY. **c** P45bX0. **d** P45bXY. Color-coded bar plots were generated from RepeatMasker.align files after selection of all TE copies with K2P distances < 5%. The graphs represent the base pairs occupied by a given TE superfamily (*y* axis) in the different genomes analyzed, binned according to K2P distances to their corresponding consensus sequence (*x* axis, K2P distance from 0 to 5%). We considered copies in the divergence bins < 5% as recent TEs, likely corresponding to copies of recently active elements. Plots showing all divergence bins (K2P distance from 0 to 50%) are shown in Additional file 2: Figure S2

Assuming that the K2P distance from the consensus sequence reflects the time since the insertion of a TE copy, this can be a proxy for the temporal accumulation of TEs [76]. Based on this assumption, we quantified the accumulation of recent TEs that were between 0 and 5% diverged from the respective consensus sequence (Fig. 3). We found a total length of 314–464 Mb (6.98–10.01% of genomes) of TEs with 0–5% divergence in each assembly, suggesting massive recent amplifications of the five major TE groups in each race (Table 4). This is larger than the estimated genome size of *Drosophila* and many other dipterans, coleopterans, lepidopterans, hymenopterans, and hemipterans, for example [54].

**Table 4:** Assembly-based quantification of the accumulation of recent TEs that were between 0 and 5% diverged from the respective consensus sequence. Total megabase and density of different classes of TEs annotated by RepeatMasker using a combined library of the RepeatModeler de novo library from each race (manual curation of the P24X0 library, used for reclassification of the three other de novo libraries), the Arthropod library from Repbase, and libraries from HelitronScanner and RepeatExplorer2, across males of four chromosomal races of the *viatica* species group. *Mb* megabase, *GP* genome proportion (% assembly)

Repeat type	P24X0		P24XY		P45bX0		P45bXY	
	Total Mb	GP	Total Mb	GP	Total Mb	GP	Total Mb	GP
SINE	7	0.16	2	0.04	7	0.15	14	0.28
LINE	126	2.96	97	2.16	158	3.41	122	2.41
LTR	42	0.98	25	0.56	56	1.21	36	0.71
DNA	175	4.11	167	3.71	200	4.31	181	3.57
Helitron	35	0.82	23	0.51	43	0.93	47	0.93
**Total**	**385**	**9.03**	**314**	**6.98**	**464**	**10.01**	**400**	**7.9**

We considered the K2P distance bins of 0 to 1% as very recent TEs that likely accumulated during or after divergence of the chromosomal races. Some of the most abundant TE superfamilies with very recent copies were DNA/DNA, DNA/P, DNA/Sola, DNA/hAT, DNA/TcMar, Helitron, LINE/L2, LTR/LTR, LTR/Gypsy, and SINE/tRNA (Fig. 3). The proportions of these TE superfamilies in each assembled genome were different in this divergence bin, with largest variation for LTR/Gypsy (e.g., P24XY = 0.16%, P24X0 = 0.33%, P45bXY = 0.34%, P45bX0 = 0.71%) and DNA/TcMar (e.g., P24XY = 0.09%, P24X0 = 0.37%, P45bX0 = 0.67%, P45bXY = 0.67%) indicating differential amplification.

### Transcriptional activities of TEs

We next characterized TE expression in the P24X0 and P24XY races (5–11 individuals per tissue/sex/race) by comparing the number of RNA-seq reads mapping to recent TE copies (i.e., copies with K2P distance < 5%; hereafter referred to as “recent TE expression” data), using RepEnrich2 (https://github.com/nerettilab/RepEnrich2) and DESeq2 1.20.0 [77]. For comparison, we repeated the analysis with all TE copies regardless of K2P distance. For this analysis, we provided RepEnrich2 with a filtered RepeatMasker annotation file containing only TE loci with < 5% K2P from the consensus sequences. This step was required to survey recent TE expression because RepEnrich2 does not retain locus coordinates, preventing us to subsample recent TE expression if the total TE expression is considered (i.e., including older TE copies with K2P > 5%).

**Fig. 4:**
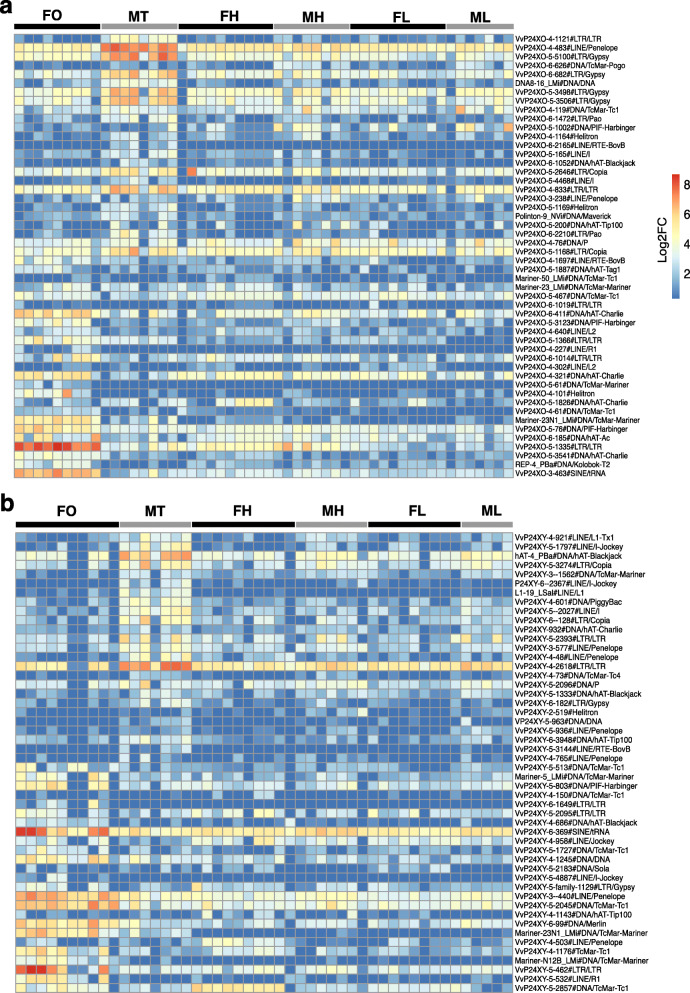
Heatmaps showing RNA-seq expression of the 50 most highly expressed TE subfamilies across sexes and tissues of two chromosomal races of the *viatica* species group. **a** P24X0. **b** P24XY. Expression levels shown as log2-normalized counts (log2 fold-change, *P* < 0.05). The color-coded bar indicates the expression levels in each sex and tissue. Each row represents a TE consensus sequence (i.e., subfamily) and each column the biological replicates. The analysis represents recent TE expression based on the TE copies with K2P < 5% of divergence to their corresponding TE consensus sequence. FO = female ovary; MT = male testis; FH = female head; MH = male head; FL = female leg; FM = male leg

In the analysis of recent TE expression, there were 598 and 1415 expressed TE subfamilies after DESeq2 normalization (by removal of low-count elements) in the reproductive tissues (male testis and female ovary) of P24X0 and P24XY, respectively. Of these, we observed a higher proportion of female-biased TEs (FBTEs) relative to male-biased TEs (MBTEs) in P24X0 (FBTEs 15.05%, MBTEs 10.37%); P24XY showed the opposite trend between sexes (MBTEs 4.88%; FBTEs 3.39%) (Table 5). We found 574 and 1415 expressed TE subfamilies in head tissues of P24X0 and P24XY after DESeq2 normalization, respectively. Of these, we observed a higher proportion of MBTEs relative to FBTEs in P24X0 (MBTEs 3.66%, FBTEs 0.35%) and P24XY (MBTEs 1.48%, FBTEs 0.21%) (Table 5). In leg tissues, a total of 1362 and 775 expressed TE subfamilies were observed after DESeq2 normalization in P24X0 and P24XY, respectively. There was not much variation in TE expression levels in leg between sexes in both races: P24XY (MBTEs 0%, FBTEs 0.52%) and P24X0 (MBTEs 0.23%, FBTEs 0.07%). The heatmaps showing the expression data of the 50 most highly expressed TE subfamilies in three tissues contain representatives from all five major TE groups (Fig. 4). The numbers of differentially expressed TEs between sexes and tissues when including all TE copies regardless of K2P distance were much larger (between 2354 and 7240 TEs transcribed; Additional file 1: Table S1) than the recent TE expression data, likely because of transcription of old and inactive TE copies.

**Table 5:** Differentially expressed TE subfamilies containing only recent TEs (genomic copies with < 5% K2P distance from the consensus sequences) between sexes and tissues in two chromosomal races of the *viatica* species group

Race	Sample	No. of expressed TEs	SBTEs (%)	MBTEs (%)	FBTEs (%)
P24X0	Head	574	23 (4.05)	21 (3.66)	2 (0.35)
	Gonad	598	152 (25.42)	62 (10.37)	90 (15.05)
	Leg	1362	4 (0.29)	3 (0.23)	1 (0.07)
P24XY	Head	1415	24 (1.71)	21 (1.48)	3 (0.21)
	Gonad	1415	117 (8.27)	69 (4.88)	48 (3.39)
	Leg	775	4 (0.52)	0	4 (0.52)

We report the total number of TEs expressed, and those TEs that were sex-biased with log2FC > 0 and log2FC < 0 and adjusted *P* < 0.05; log2FC = log2 fold-change. *SBTEs* sex-biased TEs, *MBTEs* male-biased TEs, *FBTEs* female-biased TEs

### Tandem repeat (TR) identification and sequence characterization

Since TRs were underrepresented in the aforementioned linked-read assemblies, we applied two read-based approaches, RepeatExplorer2 [72–74] and NOVOplasty 3.7.2 [75], and merged the outputs of these two to detect TRs in each race. We identified 56 TR families in P24X0, 60 TR families in P24XY, 71 TR families in P45bX0, and 92 TR families in P45bXY. These TRs included multigene families (45S rRNA, 5S rRNA, U snRNA, and histones genes), the tandem telomere repeat (TTAGG)n, and satDNA families. None of the TRs were found in the aforementioned RepeatModeler libraries. The total abundance of TRs composed of multigene families, telomere repeats, and satDNAs represented 7.82%, 6.06%, 7.53%, and 8.85% of the sequenced reads of P24X0, P24XY, P45bX0, and P45bXY, respectively (Table 3, Additional file 1: Table S2-S5). Compared to these read-based TR quantifications, the abundance of TRs in the genome assemblies was much lower (i.e., << 0.1% per assembly), suggesting that most satDNA copies were collapsed during the assembly process. Read-based approaches thus proved essential to the identification and quantification of TRs (Tables 2 and 3).

**Fig. 5:**
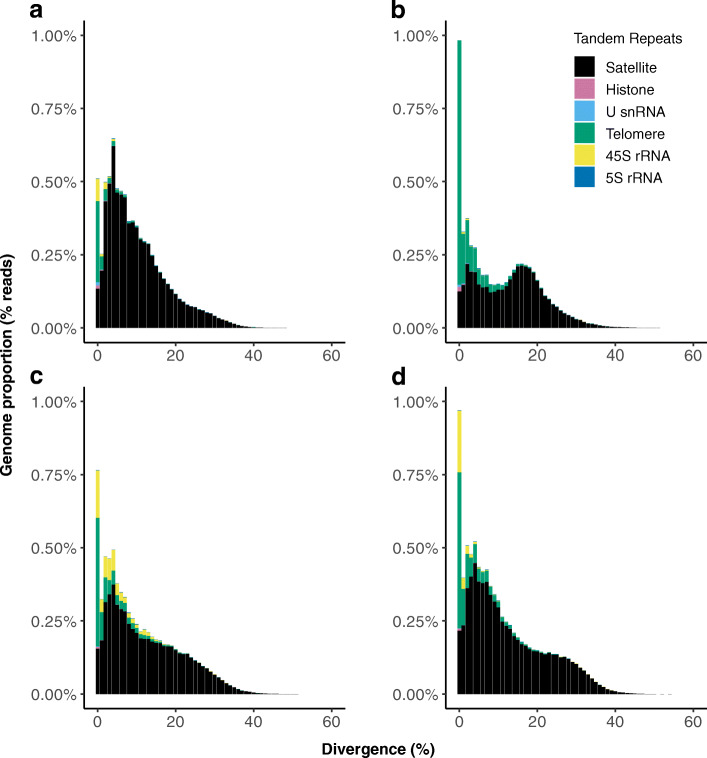
Repeat landscapes of the main groups of tandem repeats (TRs) detected in the sequenced reads of the four chromosomal races of the *viatica* species group. **a** P24X0. **b** P24XY. **c** P45bX0. **d** P45bXY. Color-coded bar plots are based on RepeatMasker showing K2P distance to their corresponding consensus sequence on the *x* axis and the TR abundance on the *y* axis. Color-coded bar plots show the proportions of TRs classified as satellite (45–81 families), telomere repeat, and the multigene families U snRNA genes (U1, U2, U5, U6), histone genes (H1, H2A, H2B, H3, H4), and 45S rRNA and 5S rRNA genes. More detailed repeat landscapes of all TR families are shown in the Additional file 2: Figure S3

The telomere repeat was more abundant in the XY races (P24XY and P45bXY, 1.61% and 1.21% of the sequenced reads, respectively) than the X0 races (P24X0 and P45bX0, < 0.91% of the sequenced reads) likely through independent amplification of the telomere repeats in the two former (Fig. 5). The 45S rRNA gene underwent slightly more differential amplification in P24XY (abundance 0.61%) than the other three races (abundances < 0.31%). Most copies of the telomere repeat and the 45S rRNA gene were in the divergence bins of K2P < 5%, likely reflecting functional constraints on their sequences.

The satDNAs were the dominant TRs in all four races (Fig. 5). RepeatExplorer2 found 45 satDNA families in P24X0 (7% of sequenced reads), 48 satDNA families in P24XY (4% of sequenced reads), 60 satDNA families in P45bX0 (6% of sequenced reads), and 81 satDNA families in P45bXY (7% of sequenced reads) (Additional file 1: Table S2-S5; Additional file 2: Figure S3). The repeat unit length (monomers) of the satDNA families ranged from 21 to 1740 bp in P24X0, from 7 to 1690 bp in P24XY, from 13 to 1710 bp in P45bX0, and from 13 to 1250 bp in P45bXY. Multiple sequence alignments and dotplots showed that most satDNA families were present in multiple contigs within the clusters of the RepeatExplorer2 results, with monomers differing by at least one nucleotide from each other, indicating that distinct sequence variants of monomers and higher-order repeat structures (HORs) were present in each genome (Additional file 1: Table S2-S5; Additional file 2: Figure S4).

#### Assessment of the satDNA library hypothesis

**Fig. 6:**
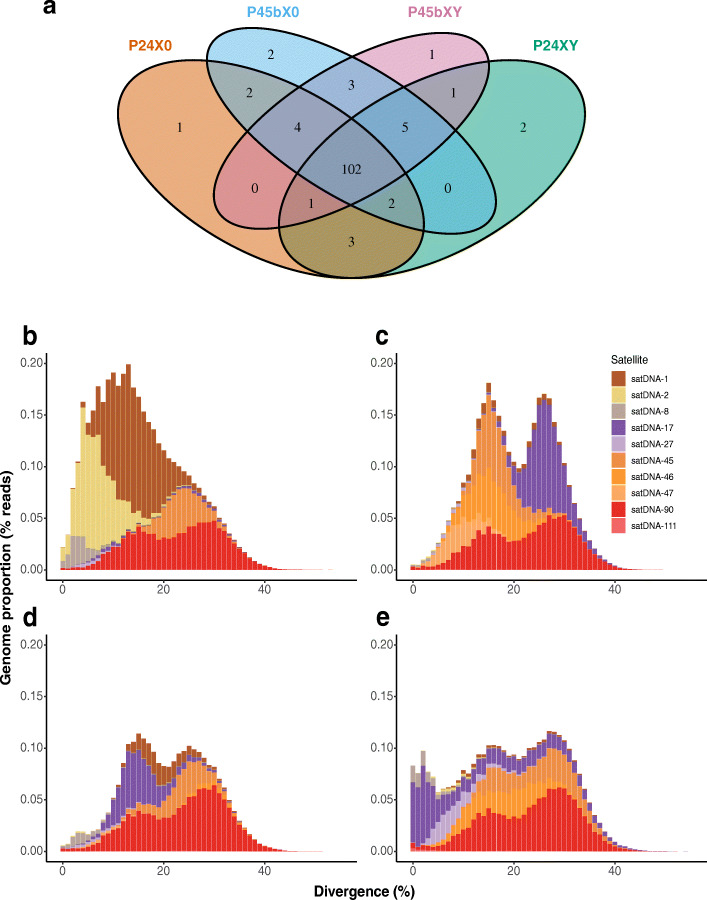
satDNA presence and divergence across four chromosomal races of the *viatica* species group. **a** Venn diagram showing the number of satDNA families shared among the chromosomal races. **b**-**e** Repeat landscapes based on RepeatMasker illustrating the abundance and divergence of 10 randomly selected satDNAs. **b** P24X0, **c** P24XY, **d** P45bX0, and **e** P45bXY. Color-coded bar plots showing the genome proportion (*y* axis) for each type of satDNAs in the sequenced reads analyzed, binned according to K2P distances to their corresponding consensus sequence (*x* axis, *K2P* value from 0 to 50%). Copies in low-divergence bins are very similar to the consensus sequence and likely correspond to very recent copies. Out of the 102 satDNA families shared between all four races, there were 50 satDNA families showing differential amplification in copy number (cv > 80%; Additional file 1: Table S8). From these 50 families, 10 randomly selected satDNAs are shown here for visualization of differential proliferation of satDNAs

Because RepeatExplorer2 can only detect satDNAs with abundance > 0.01% of the genome [72–74], homologous satDNAs present below this threshold can remain undetected across samples. Thus, we used two approaches to search for homologous satDNAs between races. First, we built a satDNA database by concatenating all the satDNA consensus sequences detected in each race (totaling 234 consensus sequences), and performed an all-against-all comparison of the consensus sequences in the database using the rm.homology.py script (https://github.com/fjruizruano/ngs-protocols) [36]. Second, we mapped the genomic reads of each race separately to the satDNA database using RepeatMasker. These approaches defined 129 satDNA families across the four races (hereafter referred to as satDNA-1 to satDNA-129). Out of the 129 satDNAs, 102 satDNAs were shared between all four races, and 27 satDNAs were present or absent in one or more race (Additional file 1: Table S6-S7). From these 27 satDNA families, there were 1–2 satDNA families specific to just one race, the largest number in P24XY and P45bX0 (Fig. 6a).

From the 102 satDNA families that were shared between all four races, there were 50 satDNA families that showed cv > 80% (cv min 82% and cv max 199%) for abundance data between races (Additional file 1: Table S8), indicating differential amplification since the divergence of the chromosomal races. From these 50 satDNA families, we randomly selected 10 families to generate a repeat landscape of their relative genomic read abundance in 1% bins of K2P distance. Differential amplification indicated by the differences in abundance and K2P distance distribution of sequences was significant for eight satDNAs (Kruskal-Wallis test *P* < 0.03; satDNA-1, satDNA-2, satDNA-8, satDNA-17, satDNA-27, satDNA-45, satDNA-47, satDNA-90) but not for the remaining two (*P >* 0.33; satDNA-46 and satDNA-111) (Fig. 6, Table 6). Among these 10 satDNAs, the most abundant family was satDNA-1 in P24X0 (1.48%) and the least abundant one was satDNA-47 in P24X0 (<< 0.01%). The most divergent family was satDNA-45 in P45bXY (average K2P 25.53%) and the least divergent one was satDNA-8 in P45bXY (average K2P 4.01%) (Table 6).

**Table 6:** Abundance and average divergence of the 10 satDNA families shown in Fig. 6

Repeat	Abundance (%)				Divergence (% K2P)				Differential copy amplification (Kruskal-Wallis test)
	P24X0	P24XY	P45bX0	P45bXY	P24X0	P24XY	P45bX0	P45bXY	
satDNA-1	1.48	0.13	0.23	0.06	13.94	19.60	20.28	19.86	*P* < 0.03
satDNA-2	0.96	0.01	0.01	0.02	7.12	9.50	11.31	10.18	*P* < 0.01
satDNA-8	0.15	0.01	0.06	0.08	5.10	4.72	6.57	4.01	*P* < 0.01
satDNA-17	0.06	0.50	0.57	0.75	24.4	15.77	19.48	19.90	*P* < 0.01
satDNA-27	0.02	0.03	0.03	0.19	6.73	9.51	8.44	9.29	*P* < 0.01
satDNA-45	0.39	0.53	0.32	0.51	25.33	17.38	23.58	25.53	*P* < 0.01
satDNA-46	0.01	0.51	0.05	0.44	16.64	16.42	17.12	18.30	*P* > 0.33
satDNA-47	0.01	0.27	0.03	0.01	11.57	10.01	9.83	11.14	*P* < 0.01
satDNA-90	1.20	1.05	1.20	1.20	22.82	23.02	23.85	23.67	*P* < 0.01
satDNA-111	0.004	0.01	0.005	0.02	22.46	15.34	22.84	9.78	*P* > 0.81

## Discussion

### Genome assembly

The genomes of four chromosomal races of the *viatica* species group that we assembled here with 2.94–3.27 Gb assembly sizes are the third largest assembled insect genomes so far, with the largest being the two locust grasshoppers *L. migratoria* and *Schistocerca gregaria* with 6.5 and 8.6 Gb assembly sizes, respectively [55, 56]. We believe that genome size estimates based on k-mer analysis better represent the genome size of these grasshoppers than the genome assembly sizes because highly repetitive sequences (e.g., centromeres, telomeres, satDNAs, non-recombining part of sex chromosomes) are likely collapsed during the assembly process [66, 67, 78, 79]. However, the two different k-mer approaches yielded quite different estimates between the chromosomal races (3.30–3.82 Gb by Supernova, and 5.42–6.32 Gb by findGSE), the reasons for which remain unclear. The large differences in contig size (contig N50 29.11–35.69 kb) of the assembled genomes of the *viatica* species group, *L. migratoria* (contig N50 10.78 kb) [55] and *S. gregaria* (contig N50 12.03 kb) [56] are probably due to the difference in the DNA library preparation and sequencing methods. The *L. migratoria* genome is based on Illumina short reads (2 × 45–150 bp paired-end) with multiple insert-size libraries (i.e., four paired-end libraries ranging from 170 to 800 bp and five mate-pair libraries ranging from 2 to 40 kb) while we used linked reads (150 bp paired-end Illumina short reads with barcode information from long input DNA molecules), and the *S. gregaria* assembly used paired-end and mate-pair Illumina short reads and PacBio long reads. The scaffold N50 (158 kb) of the *S. gregaria* genome assembly (8.6 Gb) is smaller than the scaffold N50 (326 kb) of *L. migratoria* and the scaffold N50 (317 kb) that we obtained in the P24X0 race. In addition, the *S. gregaria* genome assembly is even more fragmented than the *L. migratoria* as indicated by the BUSCO scores (see [56]), indicating that assembling such large grasshopper genomes is challenging even using the combination of technologies above. Although the smaller genome assemblies of the *viatica* species group were more contiguous than the assembly of *L. migratoria*, they still contained missing and fragmented genes. This may be due to 1) large numbers of repetitive non-coding DNAs, 2) intron gigantism, 3) errors during the assembly process, 4) true missing genes, 5) failure in identifying any significant matches, and/or 6) failure in the gene prediction step to produce even a partial gene model that might have been recognized as a fragmented BUSCO match [65]. Among these, we suspect that intron gigantism and repetitive elements might be the main reasons for assembly fragmentation because the length of introns, intergenic regions, and repetitive elements in grasshoppers is much larger than that of *D. melanogaster* [55, 56], for example.

### TE dynamics and genome evolution

Approximately 66 to 72% of the genome assembly of the *viatica* species group corresponds to TEs. These are even larger than the 60 to 62% reported for the genome assemblies of *L. migratoria* [55] and *S. gregaria* [56] respectively, likely owing to the combination of methodological approaches to annotate TEs that we used. Given the deep divergence of Eumastacoidea and Acridoidea (~ 197 million years ago [38]), the two Orthopteran superfamilies to which the *viatica* species group and the locusts grasshoppers belong, respectively, these findings suggest that large repeatomes are widespread in grasshoppers. The *S. gregaria* genome assembly has 18,815 annotated genes, and the *L. migratoria* genome assembly has a similar number of annotated genes (17,307) to those recently reported in two cricket species with genome assembly sizes of 1.6 Gb (TE content 40% [80]), indicating the absence of partial or whole genome duplication events in these orthopteran lineages. Additionally, there was no evidence for such large-scale genome duplication events in the *viatica* species group because only 3–5% of BUSCO genes were duplicated. Therefore, the large genome sizes in these grasshoppers are likely due to the expansion of TEs, which has been correlated with genome size evolution across the Tree of Life [2, 81]. Indeed, we found massive recent amplification in hundreds of Mb (between 314 and 464 Mb) of the TE groups per genome assembly, an amount that is notably larger than the estimated genome size of many other insects [54, 81]. The recent amplification mainly occurred in eight TE superfamilies (DNA/DNA, DNA/P, DNA/Sola, DNA/hAT, DNA/TcMar, Helitron, LINE/L2, LTR/LTR, LTR/Gypsy and SINE/tRNA) with largest variation for LTR/Gypsy and DNA/TcMar, indicating that the recent amplifications of TE superfamilies have widely shaped the TE landscape in the genomes of the chromosomal races*.* We thus suggest that the massive proliferation of TEs combined with a slow deletion rate might contribute to the genomic gigantism in grasshoppers, as proposed for other large eukaryotic genomes [81]. In this regard, it is worth mentioning that our estimation of massive recent proliferation of TEs (314–464 Mb per assembly) is likely underestimated because low-divergence TEs are generally underrepresented in genome assemblies [66, 67].

#### Recently active TEs are permissively transcribed in gonads

We restricted our analysis to transcripts that originated from recent TEs (i.e., K2P < 5%) because these are likely to be a relevant source of transcriptional and transpositional activity. Our results demonstrated that recently active TE superfamilies from all five major TE groups (LINE/SINE/DNA/Helitron/LTR) are transcribed, suggesting that at least some of the transcribed TEs are capable of (retro)transposition. Recent TE expression tended to be differentially expressed in gonads compared to somatic tissues, similar to that reported in mammalian lineages [82]. This indicates that TEs are likely transcribed and might transpose themselves more frequently in gonads, transmitting new TE insertions to the next generation. The grasshopper ovaries and testes showed uneven expression of recent TEs, suggesting that substantial TE transcriptional variation likely exists across sexes. The expression variation of recent TEs between reproductive and somatic tissues is puzzling especially because we used somatic body parts that contained multiple types of somatic tissues, and studies on vertebrates showed that there is great variability in TE expression levels across somatic tissues [83]. TE transcription also varies temporally with gonad development which explains the transcriptome complexity of gonads as a whole in animals [82, 84]. We speculate that the expression variation might result from either global epigenetic reprograming during gametogenesis or the many more cell types/stages present in gonads than in individual somatic tissues of these grasshoppers. Alternatively, TE control might be tighter in somatic tissues, such that tight repression of TEs is important for the host and more feasible in somatic tissues (no global epigenetic reprogramming). It remains to be investigated if higher transcriptional activities of all five major TE groups, particularly in reproductive tissues, is associated with higher TE repressive mechanism activation (piwi/piRNA pathway [85–87]) to prevent the potentially deleterious effects of TE (retro) transposition in the host during global epigenetic reprogramming [85–88]. To further address this, expression analyses of genes involved in the piwi/piRNA pathway will be needed together with small RNA sequencing data.

#### The chromosomal races of *V. viatica* species group share a common collection of satDNAs which mostly experienced quantitative changes during evolution

By performing a high-quality annotation of repeats, we uncovered the largest collection of satDNA families (129) ever reported for grasshopper genomes. We propose that the 102 satDNA families shared among all four chromosomal races (Additional file 1: Table S6 and S8) represent the “library” present in the *viatica* ancestor. The 27 satDNA families that were not shared between all the four races either emerged after the divergence of the *viatica* ancestor or were lost in one or more races. This implies that the essential step in the evolution of a satDNA family might either be the acquisition of biological functions or the accumulation of sufficiently many copies to be maintained in the “library” over long evolutionary periods. How the novel satDNA families emerged remains unclear in the *viatica* species group, although unequal crossing over, intra-strand homologous recombination, gene conversion, rolling-circle replication, and transposition are possible mechanisms [11, 14–16, 18, 20, 22]. After satDNA emergence, it is logical to assume that satDNA families stochastically expand or disappear and are only maintained in the long term if they acquire a function, such as in centromeres or heterochromatin formation. To test for satDNA functionality and to determine whether satDNAs were independently acquired or lost, additional data (e.g. chromosome in situ hybridization and ChIP-seq) is needed including races/species from the other morabine grasshoppers and under a robust phylogenetic hypothesis.

In line with the satDNA library hypothesis [28, 29], 50 of the 102 satDNA families shared among all four chromosomal races experienced quantitative changes in copy number, and these happened over different K2P bins of divergence (see Fig. 6b-d), suggesting parallel amplification of satDNAs in some races or contraction/deterioration in others. The changes in copy number likely occurred by unequal crossing over, which is the mechanism that can yield changes in TR abundance, either as gains (amplifications) or losses (contractions) [89]. The large number of satDNA families in these chromosomal races is puzzling. The non-coding satDNAs have been traditionally viewed as mostly useless material capable of accumulating primarily in heterochromatin [11–13, 32, 34] until they become a too heavy load for the host genome (reviewed in [50]). It will be interesting to test whether the differential amplification of satDNAs is correlated with the amount of heterochromatin or involved in the conversion of a euchromatic chromosome into a heterochromatic one, particularly in neo-Y chromosomes which have been shown to be a trap for satDNAs in grasshoppers and crickets (see [33, 34, 90]). On the other hand, it has been suggested that differential expression of satDNAs as satRNAs can cause genomic incompatibilities in hybrids because satRNAs play critical roles in kinetochore assembly (i.e., by binding to specific centromeric proteins like CENP-A and CENP-C) [30, 31, 50, 51], heterochromatin formation [49, 52, 53] and function during cell division via siRNAs and piRNA pathways in *Schizosaccharomyces pombe*, *Drosophila*, nematodes, humans [49–53, 91]. The satDNA families identified here are thus a set of candidates for future studies on which satDNAs are located in centromeres and which are involved in heterochromatin formation of grasshoppers.

## Conclusion

We have generated the so far most contiguous genome assemblies of grasshoppers with 66–75% repeat contents using 10X Genomics linked-read sequencing*.* In-depth repeat annotation proved essential to elucidate the composition and characteristics of TEs and satDNAs in large and repetitive genomes such as grasshoppers. We showed a massive recent proliferation of a wide range of TEs, many of which are transcribed more frequently in germline than somatic tissues. In addition, we uncovered the largest number of satDNA families ever reported for grasshopper genomes, and showed that, despite the recent divergence of the four *viatica* chromosomal races, satDNA evolution underwent rapid expansions or contractions in copy number. Although the difference in the extent of repeat expansion/contraction may be related to demographic history [92, 93], it may have had a critical role in the evolution of the distinct karyotypes. Both TEs and satDNAs affect sex chromosome evolution and differentiation after recombination is halted, TEs likely affecting the formation of satDNAs and the conversion of euchromatic chromosomes into heterochromatic ones [11, 40–46]*.* Finally, the evolutionary young origin of diverse karyotypes, together with multiple emergences of neo-sex chromosomes place the morabine grasshoppers of the *viatica* species group in a pivotal position to address the impact of the repeatome on the evolution of different genomic architectures. In this regard, it will be essential to use long-read sequencing technologies, such as Pacific Biosciences and Oxford Nanopore, and chromosome conformation capture (Hi-C) to generate high-quality chromosome-level assemblies in these grasshoppers with large and repetitive genomes.

## Methods

### Taxon sampling, DNA and RNA extraction, and sequencing

Chromosomal races of *Vandiemenella* morabine grasshoppers were collected between 2002 and 2017 in South Australia. To identify the races, the testes were dissected from males and fixed for karyotyping as described previously [58, 94]. The remaining body parts were flash-frozen in liquid nitrogen and stored at − 80 °C in the Australian Biological Tissue Collection until subsequent DNA extraction. One male per race (P24X0, P24XY, P45bX0 and P45bXY) was used for DNA extraction from either heads or legs using the MagAttract HMW DNA Kit (QIAGEN, Hilden, Germany; Cat No. 67563). Sequencing libraries were prepared as recommended by Chromium Genome preparation kit (10X Genomics, Inc., Pleasanton, CA, USA; Cat No. 120215). Paired-end reads sequencing (2 × 150 bp) was performed on the Illumina HiSeq X (Illumina, Inc., San Diego, CA, USA) using the Chromium library.

Tissues from males (head, leg, and testes) and females (head, leg, and ovary) of the chromosomal races P24X0 and P24XY were dissected and placed in RNAlater (Thermo Fisher Scientific, Waltham, MA, USA) and stored at − 80 °C until subsequent RNA extraction. In total, 42 males and 62 females were used for RNA extraction (5–11 individuals per tissue/sex/race). We extracted RNA with phenol-based phase separation using the TRIzol reagent (Thermo Fisher Scientific) following the standard protocol recommended by the supplier. Sequencing libraries were prepared according to the TruSeq stranded mRNA library preparation kit (Illumina, Inc., Cat No.20020594/5) including poly-A selection. Paired-reads (2 × 100 bp) were sequenced on the NovaSeq 6000 S2 flowcell (Illumina, Inc.). DNA and RNA library preparation and sequencing were performed at the SNP&SEQ Technology Platform in Uppsala, Uppsala University, Department of Medical Sciences, Uppsala Biomedical Centre (BMC) (Uppsala, Sweden).

### Genome size estimation and genome assembly

We performed genome size estimation by counting k-mer frequency of the quality checked 10X Genomics linked reads. The multiple fastq.gz files were two-step processed for counting *k*-mers using Jellyfish 2.2.6 [95] with the following setting: -t 8 -C -m 18 -s 5G --min-quality=20 --quality-start=33. We used the R package findGSE [68] to estimate genome sizes by using the output of Jellyfish.

We used Supernova 2.1.0 [64] to generate a “pseudohaploid” genome assembly of males of P24X0, P24XY, P45bX0, and P45bXY using 10X Genomics linked-read data. Supernova uses the 10X Genomics linked reads generated from a single library of DNA from an individual organism as source, potentially allowing for the assembly of longer contigs and scaffolds than conventional short-read technologies [64]. For comparative analysis of the generated draft assemblies, we downloaded the *Locusta migratoria* genome from LocustBase (http://159.226.67.243/download.htm). The completeness of the assemblies was evaluated with BUSCO v3 [65] with the Arthropoda database [96] as a reference. The recovered matches were classified as complete if their lengths were within the expectation of the BUSCO profile match lengths. If these were found more than once, they were classified as duplicated. The matches that were only partially recovered were classified as fragmented, and BUSCO groups that passed the test of gene prediction but for which there were no matches in the database were classified as missing.

### Assessing the TEs content across genomes

We used the draft male genome assemblies of P24X0, P24XY, P45bX0, and P45bXY to generate repeat libraries for each of the four genomes using RepeatModeler 1.0.8 [69]. Because the P24X0 had the highest assembly contiguity and BUSCO scores, the repeats classified as unknown in the P24X0 RepeatModeler library were selected for manual curation following the method used in Suh et al. [97]. Every consensus sequence was aligned back to the assembled genome sequence of P24X0, then the best 20 BLASTn hits were collected, extended by 2 kb and aligned to one another using MAFFT 7 [98]. Manually curated consensus sequences of P24X0 were then classified as TE families/subfamilies based on the proposed classification system for TEs (open reading frames, terminal repeats, target site duplications) [7] and Repbase similarity searches [99]. We removed redundancies from the curated TE consensus sequences of P24X0 by merging sequences that were greater than 80% similar using CD-HIT-EST [100], with the following setting: -c 0.80 -n 5 -M 0 -aS 0.80 -G 0 -g 1. The curated TE library from P24X0 race was used as a reference to reclassify repeats in the TE libraries of the other three races (i.e., P24XY, P45bX0, and P45bXY) by using RepeatMasker 4.0.8 [70] for homology search. We used RepeatMasker because it outperformed a BLASTN homology search, especially for satDNAs where homology for only 16 families was found. We then manually inspected the RepeatMasker hits to corroborate that they were indeed homologous across most of the length of a single consensus (following the 80-80-80 rule) [7]. We then removed redundancies of the reclassified TEs within each race-specific library using CD-HIT-EST.

In addition to the annotation of TEs in the four RepeatModeler libraries above, we also searched for new Helitrons across the genome assemblies using HelitronScanner 1.0 [71]. HelitronScanner uses the two-layered local combinational variable (LCV) tool for Helitron identification. To avoid false positive in Helitron searches, we used thresholds of 7 for both Helitron ends (14 for sum of LCV scores) as a parameter in the HelitronScanner runs. We used CD-HIT-EST to remove redundancies of the detected Helitron sequences by merging sequences that were greater than 80% similar. This produced a set of non-redundant representative sequences (families) of new Helitrons. We used the detected Helitrons in each chromosomal race to finally mask the respective genomes with RepeatMasker using a race-specific combined repeat library (race-specific libraries for RepeatModeler + HelitronScanner + RepeatExplorer2 combined with Repbase Arthropoda repeats) (see “Tandem repeat detection in sequenced reads” section for the RepeatExplorer2 library).

We then used each of the .align RepeatMasker output files per genome assembly to estimate abundance and divergence of each TE superfamily. To visualize the temporal activity/accumulation of TEs across races, we generated landscape bar plots depicting the relative abundance of repeat elements on the *y* axis and the K2P distance from the respective consensus sequence on the *x* axis. The K2P distance model corrects for multiple hits, taking into account two-state transitions (transitional and transversional substitution rates), while assuming that the four nucleotide frequencies (A, T, C, and G) are the same and that substitution rates do not vary among sites [101].

We further quantified the repeatome between races using read-based approaches to compare with the assembly-based approaches. For this purpose, the relative genomic abundance of repeats in the sequenced reads were compared by random sampling of 4 million read pairs per library and masking them with the race-specific combined repeat library (see previous paragraph) using RepeatMasker. This quantification was done separately in each race using their respective repeat library. TRs were detected using RepeatExplorer2 [72–74] and NOVOplasty 3.7.2 [75] (see below).

### Transcription of TEs

We used Illumina RNA-seq reads (2 × 100 bp) from males and females (testis, ovary, head, and leg) of P24X0 and P24XY races to investigate the transcriptional profile of TEs in each tissue. For comparative purposes, we used the RepeatMasker annotation (with simple and low complexity repeats removed) from the P24X0 and P24XY races to check whether the TEs show differential expression among sexes and tissues and to compare the intensity and direction of the bias among races. RNA-seq reads per sample were trimmed with TrimGalore (https://github.com/FelixKrueger/TrimGalore)*.* Erroneous k-mers from Illumina paired-end reads were removed using rCorrector [102]*.* Unfixable reads (often riddled with N nucleotides or represented by other low complexity sequences) were discarded using FilterUncorrectablePEfsta.py script obtained from the Harvard Informatics GitHub repository (https://github.com/harvardinformatics/TranscriptomeAssemblyTools). We used SortMeRNA 3.0.3 [103] for local alignment, filtering, mapping, and clustering to remove rRNA.

We applied the RepEnrich2 protocol (https://github.com/nerettilab/RepEnrich2) to estimate TE transcript expression levels in each genome assembly. To assign mapped reads to a genomic locus, RepEnrich2 requires an annotation file that specifies repeat element coordinates. The RepeatMasker annotation was used for the approach. We estimated the expression levels of reads that originated from recent TE copies. For this analysis, we provided RepEnrich2 with a filtered RepeatMasker annotation containing only TE copies with < 5% K2P from the consensus sequences. The filtered RepeatMasker annotation was then used to build the final annotation required for RepEnrich2 protocol by running the RepEnrich2_setup script. Next, the pre-processed RNA-seq reads were mapped to each of the assemblies using Bowtie2 2.2.9 [104] as the read mapper. The quantification step included raw read count estimated by the RepEnrich2 protocol, followed by normalization and differential expression analysis of TEs estimated by the DESeq2 1.20.0 [77]. The data regarding sex-biased TE expression were compared between gonads, heads, and legs from males and females of the chromosomal races P24X0 and P24XY. The statistical analysis of differentially expressed TE was performed using DESeq2 as implemented in the Bioconductor package [105] in R [106]. All *P* values were adjusted using the Wald test as implemented in DESeq2. A TE was considered biased if the comparison for the factor condition (samples) yielded an adjusted *P* < 0.05. Since the factor condition was the samples and different races were analyzed, we performed phylogenetic independent contrasts. The degree of bias was determined by the log2 fold-change (log2FC) difference between conditions as calculated in DESeq2, i.e., those TEs with log2FC > 0 and log2FC < 0 and with an adjusted *P* < 0.05 were considered as male-biased TE (MBTEs) and female-biased TE (FBTEs), respectively.

### Tandem repeat detection in sequenced reads

We used RepeatExplorer2 [72–74] to identify satDNAs using the linked reads of each chromosomal race used for genome assembly above. RepeatExplorer was run separately in each race. Prior to RepeatExplorer2 graph-based clustering analysis, sequence reads were quality-trimmed using TrimGalore. The trimmed paired-end reads were joined by using the “fastq-join” software of the FASTX-Toolkit suit [107] with default parameters. The joined paired-end reads were then subject to graph-based clustering and assembly using RepeatExplorer2. A set of randomly selected 2,000,000 Chromium linked reads with average length of 150 bp were used as an input for clustering analysis.

We used the dotplot graphic alignment tool implemented in Dotlet [108] to confirm the tandem organization of those clusters with high graph density in RepeatExplorer2 output. Additionally, we used the FlexiDot 1.06 [109] suite to generate all-against-all dotplot visualization of those clusters identified as tandem arrays. The monomer with maximum length was used as the representative copy for each satDNA family, and as the query sequences in further BLASTn (http://www.ncbi.nlm.gov/Blast/) and Repbase [99] searches to check for similarity with published sequences, and as a query to check for overlap with the RepeatModeler libraries. We named each satDNA family as Vv (from *Vandiemenella viatica* group) followed by the provisional taxon name (i.e., P24X0, P24XY, P45bX0, and P45bXY) and a number in descending order of the genome proportion. To search for HORs, we counted the maximum number of tandem monomer arrays per contig for each satDNA family by analyzing the dotplots. We then counted the total number of monomers present in each cluster. The multiple sequence alignments of satDNA copies were generated using Muscle [110] implemented in MEGA 5 [111].

We ran NOVOPlasty 3.7.2 [75] to search for tandem multigene families, such as 45S rRNA, 5S rRNA, U snRNA (U1, U2, U5, U6), and histone genes (H1, H2A, H2B, H3 and H4). We used known seed sequences of these genes coming from other species and k-mer between 21 and 23 as parameters for assembly: Gomphocerinae sp. for the 45S rRNA gene (AY859546.1), *Ronderosia bergii* for the 5S rRNA gene (KP213274), *Nasonia vitripennis* for the H1 histone gene (XM_003423983.3), *Culex quinquefasciatus* for the H2A (XM_001862631.1) and H2B (XM_001870471.1) histone genes, *Locusta migratoria* for the H3 histone gene (GU111931.1), *N. vitripennis* for the H4 histone gene (XM_001599180.3:49-357), *Eyprepocnemis plorans* for the U1 snRNA gene (KJ606069.1), *Abracris flavolineata* for the U2 rRNA gene (KP975085.1), and *D. melanogaster* for the U5 snRNA (NR_001933.1) and U6 snRNA genes (NR_002081.1). The canonical tandem telomere repeat (TTAGG)n recognized in insects [112] was recovered from the RepeatExplorer2 output.

The relative genomic abundances and K2P divergences of TRs were estimated individually in each race by sampling 4 million read pairs per library retrieved by seqtk (https://github.com/lh3/seqtk) and aligning them to the TR database with RepeatMasker. The sampled reads were mapped to dimers of satDNA consensus sequences, and for smaller TRs, several monomers were concatenated until reaching roughly 150 bp array length. To visualize the temporal accumulation of TRs across races, we generated TR landscape plots that depict the relative abundance of repeat elements on the *y* axis and the K2P distance from the respective consensus sequence on the *x* axis.

To search for homologous satDNAs between races, we used two approaches. First, we built a satDNA database by concatenating all the satDNA consensus sequences detected in each race and performed an all-against-all comparison of the consensus sequences in the database using the rm.homology.py script (https://github.com/fjruizruano/ngs-protocols) [36]. Second, we mapped the genomic reads of each race separately to the satDNA consensus database using RepeatMasker, as mentioned above, and recorded presence/absence of satDNAs in each of the four races.

## Supplementary information

**Additional file 1: Table S1.** Total numbers of differentially expressed TE subfamilies (consensus sequences) containing all TEs between sexes and tissues in two chromosomal races of the *viatica* species group. **Table S2.** Most abundant tandem repeats (TRs) in the P24X0 sex chromosome race (male reads). The repeats are sorted in descending order of the genomic read proportion. **Table S3.** Most abundant tandem repeats (TRs) in the P24XY sex chromosome race (male reads). The repeats are sorted in descending order of the male genome proportion. **Table S4.** Most abundant tandem repeats (TRs) in the P45bX0 sex chromosome (male reads). The repeats are sorted in descending order of the genome proportion. **Table S5.** Most abundant tandem repeats (TRs) in the P45bXY sex chromosome race (male reads). The repeats are sorted in descending order of the genome proportion. **Table S6.** Presence of 129 satDNA families across four chromosomal races of *viatica* species group. Homology search based on 1) all-against-all RepeatMasker comparison of satDNA consensus sequences among races, and on 2) genomic read mapping against the consensus sequences as reference in RepeatMasker. Each column represents the library of satDNAs of the race, and each row indicates the common satDNAs between them. satDNAs highlighted in gray were used as references for read mapping. The signs positive (+) and negative (−) indicate presence or absence of the satDNA family in the race based on read mapping. Columns with satDNA name non highlighted in gray indicates homologous consensus sequences between races, based on all-against-all RepeatMasker comparison of satDNA consensus sequences. **Table S7.** The number of satDNA families that were present in only one of the races or absent in at least one race. **Table S8.** Statistics of the 102 satDNA families shared across the four chromosomal races of the *viatica* species group. 50 satDNA families show differential amplification in copy number (cv > 80%). GP = Genome proportion. K2P = Kimura 2-parameter distance. sd = standard deviation. cv = coefficient of variation.

**Additional file 2: Figure S1.** Comparison of the k-mer distributions (18-mer frequency) in the genomic reads of four chromosomal races of the *viatica* species group. a) P24X0. b) P24XY. c) P45bX0. d) P45bXY. The numbers of k-mers (18-mer frequencies) were plotted against the 18-mer coverage. The peak coverage (k-mer cov) corresponding to heterozygous k-mers (heterozygosity) as well as the repetitive content are indicated in the plot legends. **Figure S2.** Comparison of transposable element landscapes in male genome assemblies of four chromosomal races of the *viatica* species group. a-h) Percentage of bp occupied in the genome (*y* axis) plotted against the Kimura 2-parameter (transitions/transversions) distance (*x* axis) of copies from each TE superfamily (color-coded) from their consensus sequences. a,c,e,g) Based on *de-novo* predicted repeats from RepeatModeler (RML) and Arthropoda Repbase library (*A*RL) repeats. b,d,f,h) Based on curated *de-novo* predicted repeats (curation of the P24X0 library used for re-classification of the three other *de-novo* libraries) from RepeatModeler + *A*RL repeats. Note the share of unknown (gray) repeats, a majority of which were identified as LTR retrotransposons (green) and DNA transposons (orange/red) when manually curated. **Figure S3.** Tandem repeat (TR) landscapes in the male sequenced reads of four chromosomal races of the *viatica* species group. a) P24X0. b) P24XY. c) P45bX0 d) P45bXY. Temporal accumulation of TRs is shown as repeat element divergence in Kimura-2 parameter (K2P) distance to consensus on the *x* axis and the TR abundance in on the *y* axis. The satDNAs are named as “Vv” (for *Vandiemenella viatica* group) followed by “P” (for provisional taxon) and a number that indicates the family number in decreasing order of the genomic read proportion of the race. **Figure S4.** All-against-all dotplot comparisons showing the diversity of satDNAs arrays detected among four chromosomal races of the *viatica* species group. a) The VvP24X0-6 (28 bp) satDNA in the P24X0 race. b) The VvP24XY-6 (167 bp) satDNA in the P24XY race. c) The VvP45bX0-50 (171 bp) satDNA in the P45bX0 race. d) The VvP45bXY-11 (52 bp) satDNA in the P45bXY race. The satDNAs are named as “Vv” (for *Vandiemenella viatica* group) followed by “P” (for provisional taxon) and a number that indicates the family number in decreasing order of the genomic read proportion of the race. Each satDNA family was defined by graph-based clustering of sequenced reads in RepeatExplorer2 (see Materials and Methods section). For simplicity, the plot shows all-against-all comparisons of the first six contigs (C1-C6) within the cluster. Contigs are aligned against themselves, against one another, and against their monomer consensus sequence (Cons). The different gray/black shades enable the identification of long shared subsequences between contigs at a glance, based on longest common subsequence, or longest match if mismatches are considered. Longer matches are represented by darker background shading.

**Additional file 3.** Repeat consensus sequences of the P24X0 sex chromosome race (male) of the of the viatica species group (tar.gz file).

**Additional file 4. **Repeat consensus sequences of the P24XY sex chromosome race (male) of the of the viatica species group (tar.gz file).

**Additional file 5. **Repeat consensus sequences of the P45bX0 sex chromosome race (male) of the of the viatica species group (tar.gz file).

**Additional file 6. **Repeat consensus sequences of the P45bXY sex chromosome race (male) of the of the viatica species group (tar.gz file).

## Data Availability

The datasets generated as part of the study are available as supplementary information. Repeat consensus sequences are available as Additional file 3 (P24X0), Additional file 4 (P24XY), Additional file 5 (P45bX0), and Additional file 6 (P45bXY). Each of these additional files contains libraries generated by RepeatModeler (including curated and reclassified repeats), HelitronScanner, and RepeatExplorer2. Raw reads and assemblies were deposited in NCBI under the BioProject PRJNA668746.

## Abbreviations

A+T%: Percentage of A + T bases, i.e., the molar ratio of adenine and thymine in DNA
cv: Coefficient of variation
HOR: Higher-order repeat structure
K2P: Kimura 2-parameter distance
LINE: Long interspersed nuclear element
LTR: Long terminal repeat
SINE: Short interspersed nuclear element
TE: Transposable element
TIR: Terminal inverted repeat
TR: Tandem repeat
satDNA: Satellite DNA
MBTE: Male-biased transposable element
FBTE: Female-biased transposable element
log2FC: log2 fold-change
