# Supplementary material for: Comparative analysis of morabine grasshopper genomes reveals highly abundant transposable elements and rapidly proliferating satellite DNA repeats
Source: BMC Biol. 2020 Dec 21;18:199. doi: 10.1186/s12915-020-00925-x (PMC7754599; doi:10.1186/s12915-020-00925-x)
Supplement: Supplementary file 2 — Additional file 2: Figure S1. Comparison of the k-mer distributions (18-mer frequency) in the genomic reads of four chromosomal races of the viatica species group. a) P24X0. b) P24XY. c) P45bX0. d) P45bXY. The numbers of k-mers (18-mer frequencies) were plotted against the 18-mer coverage. The peak coverage (k-mer cov) corresponding to heterozygous k-mers (heterozygosity) as well as the repetitive content are indicated in the plot legends. Figure S2. Comparison of transposable element landscapes in male genome assemblies of four chromosomal races of the viatica species group. a-h) Percentage of bp occupied in the genome (y axis) plotted against the Kimura 2-parameter (transitions/transversions) distance (x axis) of copies from each TE superfamily (color-coded) from their consensus sequences. a,c,e,g) Based on de-novo predicted repeats from RepeatModeler (RML) and Arthropoda Repbase library (ARL) repeats. b,d,f,h) Based on curated de-novo predicted repeats (curation of the P24X0 library used for re-classification of the three other de-novo libraries) from RepeatModeler + ARL repeats. Note the share of unknown (gray) repeats, a majority of which were identified as LTR retrotransposons (green) and DNA transposons (orange/red) when manually curated. Figure S3. Tandem repeat (TR) landscapes in the male sequenced reads of four chromosomal races of the viatica species group. a) P24X0. b) P24XY. c) P45bX0 d) P45bXY. Temporal accumulation of TRs is shown as repeat element divergence in Kimura-2 parameter (K2P) distance to consensus on the x axis and the TR abundance in on the y axis. The satDNAs are named as “Vv” (for Vandiemenella viatica group) followed by “P” (for provisional taxon) and a number that indicates the family number in decreasing order of the genomic read proportion of the race. Figure S4. All-against-all dotplot comparisons showing the diversity of satDNAs arrays detected among four chromosomal races of the viatica species group. a) The VvP24X0-6 (28 bp) satD [file 12915_2020_925_MOESM2_ESM.doc]

**Additional file 2**

**C****omparative analysis of morabine grasshopper genomes reveals abundance of transposable elements and extreme proliferation of satellite repeats**

Octavio M. Palacios-Gimenez^1,2^, Julia Koelman^1^, Marc Palmada-Flores^1^, Tessa M. Bradford^3,4^, Karl K. Jones^3^, Steven J. B. Cooper^3,4^, Takeshi Kawakami^1,5^*, Alexander Suh^1,2,6^*

^1^Department of Ecology and Genetics – Evolutionary Biology, Evolutionary Biology Centre, Uppsala University, SE-752 36 Uppsala, Sweden

^2^Department of Organismal Biology – Systematic Biology, Evolutionary Biology Centre, Uppsala University, SE-752 36 Uppsala, Sweden

^3^Evolutionary Biology Unit, South Australian Museum, Adelaide, SA 5000, Australia

^4^School of Biological Sciences and Australian Centre for Evolutionary Biology and Biodiversity, The University of Adelaide, Adelaide, SA 5005, Australia

^5^Embark Veterinary, Inc., Boston, Massachusetts, United States of America

^6^School of Biological Sciences, University of East Anglia, Norwich Research Park, NR4 7TU, Norwich, United Kingdom

*equal contributions

**Correspondence:** Octavio M. Palacios-Gimenez, Alexander Suh and Takeshi Kawakami, Department of Organismal Biology – Systematic Biology, Evolutionary Biology Centre, Uppsala University, SE-752 36 Uppsala, Sweden

**Emails:** octavio.palacios@ebc.uu.se (O.M.P.G.), alexander.suh@ebc.uu.se (A.S.), [tkawakami@embarkvet.com](mailto:tkawakami@embarkvet.com) (T.K.)

**Figure S1: Comparison of the k-mer distributions (18-mer frequency) in the genomic reads of four chromosomal races of the *viatica* species group. a)** P24X0. **b)** P24XY. **c)** P45bX0. **d)** P45bXY. The numbers of k-mers (18-mer frequencies) were plotted against the 18-mer coverage. The peak coverage (k-mer cov) corresponding to heterozygous k-mers (heterozygosity) as well as the repetitive content are indicated in the plot legends.


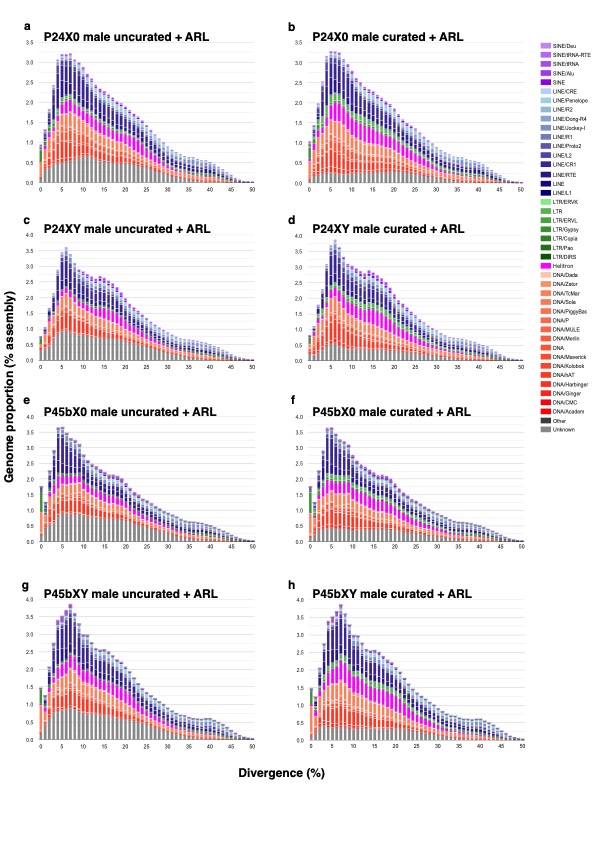


**Figure S2: Comparison of transposable element landscapes in male genome assemblies of four chromosomal races of the *viatica* species group. a-h)** Percentage of bp occupied in the genome (*y* axis) plotted against the Kimura 2-parameter (transitions/transversions) distance (*x* axis) of copies from each TE superfamily (color-coded) from their consensus sequences. **a,c,e,g)** Based on *de-novo* predicted repeats from RepeatModeler (RML) and Arthropoda Repbase library (*A*RL) repeats. **b,d,f,h)** Based on curated *de-novo* predicted repeats (curation of the P24X0 library used for re-classification of the three other *de-novo* libraries) from RepeatModeler + *A*RL repeats. Note the share of unknown (grey) repeats, a majority of which were identified as LTR retrotransposons (green) and DNA transposons (orange/red) when manually curated.


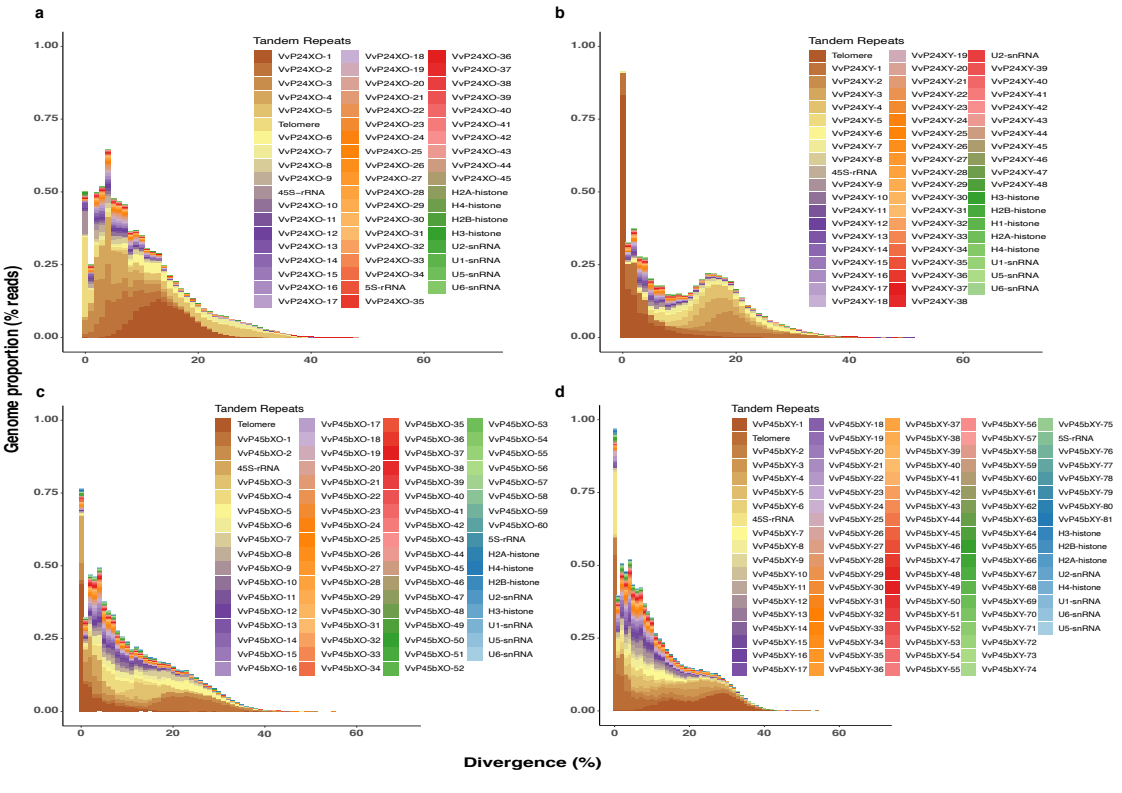
**Figure S3: Tandem repeat (TR) landscapes in the male sequenced reads of four chromosomal races of the *viatica* species group. a)** P24X0. **b)** P24XY. **c)** P45bX0. **d)** P45bXY. Temporal accumulation of TRs is shown as repeat element divergence in Kimura-2 parameter (K2P) distance to consensus on the *x* axis and the TR abundance in on the *y* axis. The satDNAs are named as “Vv” (for *Vandiemenella viatica* group) followed by “P” (for provisional taxon) and a number that indicates the family number in decreasing order of the genomic read proportion of the race.

**
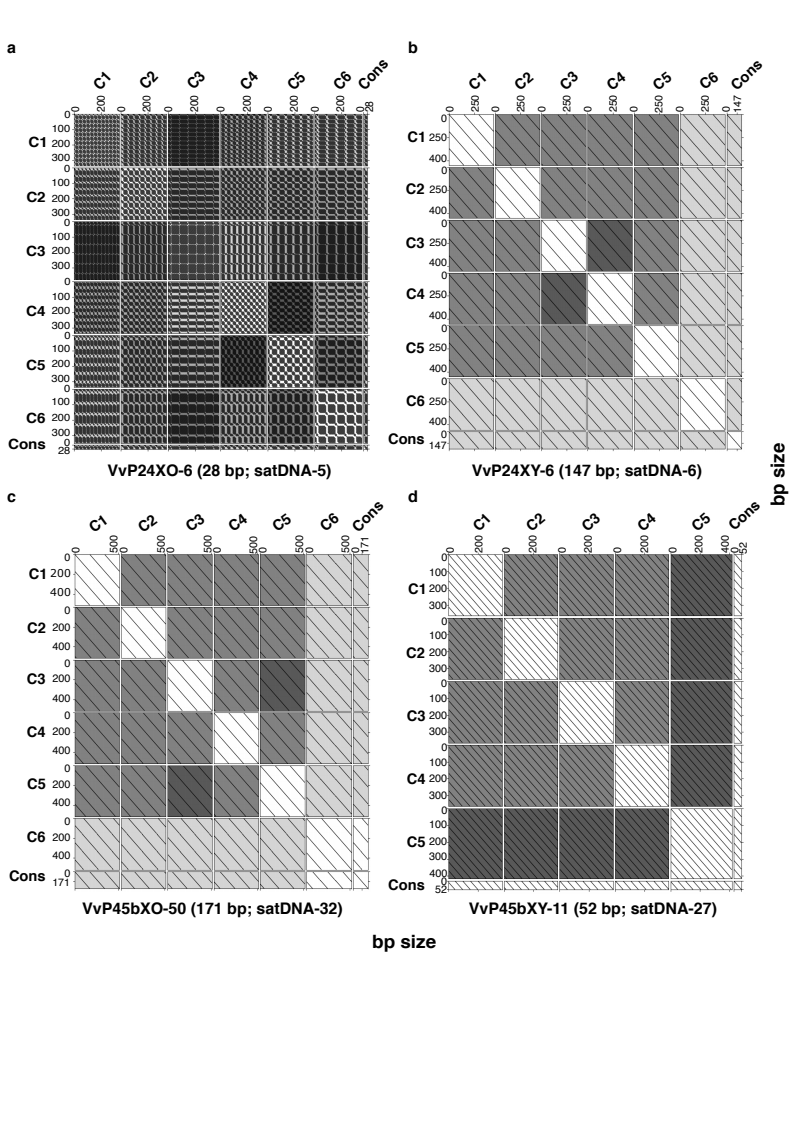
**

**Figure S4: All-against-all dotplot comparisons showing the diversity of satDNAs arrays detected among four chromosomal races of the *viatica* species group. a)** The VvP24X0-6 (28 bp) satDNA in the P24X0 race. **b)** The VvP24XY-6 (167 bp) satDNA in the P24XY race. **c)** The VvP45bX0-50 (171 bp) satDNA in the P45bX0 race. **d)** The VvP45bXY-11 (52 bp) satDNA in the P45bXY race. The satDNAs are named as “Vv” (for *Vandiemenella viatica* group) followed by “P” (for provisional taxon) and a number that indicates the family number in decreasing order of the genomic read proportion of the race. Each satDNA family was defined by graph-based clustering of sequencing reads in RepeatExplorer2 (see Materials and Methods section). For simplicity, the plot shows all-against-all comparisons of the first six contigs (C1-C6) within the cluster. Contigs are aligned against themselves, against one another, and against their monomer consensus sequence (Cons). The different grey/black shades enable the identification of long shared subsequences between contigs at a glance, based on longest common subsequence, or longest match if mismatches are considered. Longer matches are represented by darker background shading.
